# Unveiling tobacco struggle in rural areas: field insights and experiences from Rajasthan, India

**DOI:** 10.3389/fpubh.2025.1615242

**Published:** 2025-08-11

**Authors:** Harimadhav Viswanathan, Pankaja Raghav, Srikanth Srinivasan, Aswathy Suseelan Remany, Vinoth Rajendran, Pritish Baskaran, Tanya Singh

**Affiliations:** Department of Community Medicine and Family Medicine, All India Institute of Medical Sciences Jodhpur, Jodhpur, India

**Keywords:** tobacco use, rural health, tobacco cessation, stakeholder, field experiences

## Abstract

Tobacco use is a significant public health concern in rural India. This case study was conducted in a village of the Jodhpur district in Rajasthan, India. The high prevalence and patterns of tobacco use among the villagers was identified by a situational analysis in the OPD and through household visits, shopkeeper surveys, focus group discussion with health workers, and by engaging with community members, local leaders, and health workers. A general lack of adherence to Cigarettes and Other Tobacco Products Act (COTPA) rules in public places, schools, and points of sale, as well as a gap in awareness regarding the harmful effects of tobacco were found. Seven out of 10 patients attending the OPD and more than half of the village population used tobacco. Smokeless tobacco products like *mava, masheri*, *gutka*, and khaini were widely used by everyone, while older men preferred smoking forms. Children as young as 10 years old used tobacco, often encouraged by parents who believed it would suppress hunger. These findings led to the initial focus being given to comprehensive awareness through health education sessions, followed by a multistakeholder approach that engaged local leaders, police, shopkeepers, teachers, and other relevant stakeholders. Activities were conducted at health facility, administrative, and community levels. These strategies focused on awareness activities, more stringent implementation of COTPA, cessation of public display of tobacco products in shops, and the monthly celebration of ‘No Tobacco Day’. The interventions at school led to a significant improvement in the Tobacco-Free Educational Institution (ToFEI) score, from 9 pre-intervention to 90 post-intervention. The outcomes underscore that using these strategies with appropriate adaptations can be effective in tobacco control across diverse settings.

## 1 Introduction

The addiction to tobacco and its related products has remained as a major public health challenge across the globe. The extensive health effects of tobacco are not just limited to cancer and include severe illnesses affecting every vital organ (1). Around 33% of all cancer-related deaths are due to tobacco use (2). The World Health Organization (WHO) estimates that by 2030, an alarming situation will unfold. Tobacco and its related products would be causing a staggering 8 million deaths every year, and this will account for 10% of the global mortality by 2030 (2). More than 80% of global tobacco users live in Low-and middle-income countries (LMICs). Over 1.3 billion people in LMICs use one or the other form of tobacco products, resulting in a very high burden of tobacco-related illness and deaths in these parts of the world (3). The highest prevalence of tobacco use has been reported from the Southeast Asian Region (SEAR), which includes India as well (4).

The widespread use of various forms of smoking and smokeless tobacco products makes the tobacco challenge in India multidimensional and unique (5). According to the Global Adult Tobacco Survey (GATS 2), 28.6% of the Indian population above the age of 15 years uses some form of tobacco products, of which 24.9% are daily users and 3.7% are occasional users. The prevalence of tobacco use in India is higher among men (42.4%) compared to women (14.2%), and higher in rural areas (32.5%) compared to urban areas (21.2%) (6). The latest National Family Health Survey, done in 2019–20, also showed a similar pattern. The overall use of tobacco has decreased slightly, with 38% men and 8.9% of women aged 15 years and above using any tobacco products. The use was higher in rural areas (42.7% for men and 10.5% for women) compared to urban areas (28.8% for men and 5.5% for women) (7). Rajasthan is the state with the highest tobacco use prevalence among the nine states in North India. Tobacco use among men in Rajasthan is higher than the national rate, while it is lower than the national rate for women (41.9 and 6.9%, respectively) (7). The use is higher in rural areas (44.9% for men and 7.2% for women) compared to the urban areas (33.1% for men and 6% for women) (7).

India regulates tobacco through laws such as the Cigarettes and Other Tobacco Products Act (COTPA) of 2003, which enforces health warnings, bans public smoking, and restricts advertising (8). The country follows the “MPOWER” policies under the WHO Framework Convention on Tobacco Control (FCTC), aiming to reduce tobacco-related harm (9). The National Tobacco Control Programme, launched in 2007–08, supports awareness and enforcement of these measures. However, weak enforcement, low political priority, limited resources, and social normalization interfere with progress (10, 11). Effective implementation of the policies requires intersectoral collaboration, increased public engagement, capacity building and better coordination (12, 13).

This study was developed as a response to the observation of widespread tobacco use among patients attending the OPD of a Community Health Centre (CHC). Based on insights from a brief situational analysis in the health facility, local school, and community, a strategy involving all relevant stakeholders was developed. The primary aim was to create awareness and reduce the use of tobacco among the villagers through locally relevant and feasible interventions. Special emphasis was placed on improving compliance with COTPA regulations within the village and strengthening the tobacco control measures in school using the Tobacco Free Educational Institution (ToFEI) guidelines (14).

## 2 Methodology

### 2.1 Study duration

Four months (May 2022–August 2022).

### 2.2 Study setting

Located in the western part of India, Rajasthan is the largest state in the country. The Thar desert covers the northwestern part of the state and the economy is primarily agricultural and pastoral. This study was done in Keru village in the Jodhpur district of Rajasthan. Jodhpur spans over 22,850 sq. km and houses a population of more than 36 lakhs. Approximately half of the population resides in rural areas, where they are primarily engaged in agriculture, mining, and pastoral activities (15). The village of Keru is situated 25 km from the district capital. It has a total population of around 10,000 people. Approximately 30% of this population belongs to scheduled castes/tribes, and the literacy rate of the village is lower than the state and national rates (16). Keru is one of the field practice areas of the Department of Community Medicine and Family Medicine, AIIMS Jodhpur. Regular outpatient services and field activities are organized in the village by the department.

### 2.3 Situational analysis

A brief situational analysis was undertaken to identify the magnitude of the issue in the village. As a part of routine OPD care, it was made mandatory to ask every patient regarding tobacco use. This was followed by informal visits to households. The residential areas near the health facility were selected for this, considering the feasibility and time constraints of the activity. During these visits, the families were engaged in general health conversations and the topic of tobacco use within the household was also naturally included in it.

Tea shops often act as hot spots for selling tobacco products, and public smoking is very common there. Regular visits and casual conversations helped in developing rapport with various tea shop owners over time. This strategy was used to gather information about tobacco sale patterns and customer habits in the village. Direct questioning regarding the tobacco use or sale often results in resistance or non-cooperation.

### 2.4 Plan of action

#### 2.4.1 Health-facility level

##### 2.4.1.1 No tobacco day

On 31st May 2022, the activities were conducted as part of the World No Tobacco Day events. It served as an ideal opportunity to sensitize the patients, public, and hospital staff. A dedicated event was organized within the hospital premises. Awareness sessions were conducted by the interns, resident doctors and medical officer in-charge. A collective No Tobacco Day pledge was taken by the gathered crowd. The Sarpanch (Head of village governing council) recited the pledge aloud for everyone to repeat together. Following the event a baseline assessment of the health facility was done to see its adherence to the COTPA guidelines.

##### 2.4.1.2 Focus group discussion (FGD)

According to the National Programme for Prevention and Control of Non-Communicable Diseases (NP-NCD), Accredited Social Health Activist (ASHAs) are responsible for completing the Community-Based Assessment Checklist (CBAC) forms during their home visits (17). The form has questions related to tobacco use, and hence they are ideally positioned to have a fair idea of the tobacco users in their areas. The village had a total of 20 ASHAs. Nine ASHAs representing different areas of the village were invited for the FGD after fixing the venue, date, and time.

#### 2.4.2 Administrative level

##### 2.4.2.1 Involvement of the ‘No Tobacco Wing’ from district headquarters

An overview of the prevailing issues within the village was given to district-level officials of the ‘No Tobacco Wing’ during the discussion. Subsequently, they committed to conducting an awareness session in the health facility for the public and shopkeepers of the region.

The meeting was organized at the CHC after finalizing the date and time for meeting with the local leaders, hospital authorities, shopkeepers, and the health team. Initially, the team from the ‘No Tobacco Wing’ addressed the gathered public and gave awareness regarding the harmful effects of tobacco and the benefits of quitting tobacco. Information, Education, and Communication (IEC) materials related to harmful effects of tobacco were then disseminated among the public for further awareness. In addition to that, all the tobacco-selling shopkeepers were summoned for a separate meeting. Government officials explained the rules related to tobacco sales and emphasized the importance of adhering to them. Shopkeepers were aware that tobacco sales near schools and hospitals are prohibited but were largely unaware of other provisions under the acts. A stern warning was issued to the shopkeepers, emphasizing that subsequent violations would result in penalties as outlined in the COTPA guidelines.

##### 2.4.2.2 Involvement of police

A meeting was organized with the police Sub-Inspector of the area. The police officer was given a comprehensive overview of the present scenario in the village. The magnitude of the existing challenges and non-compliance with COTPA guidelines was pointed out. The discussion shed light on various issues leading to widespread tobacco usage in the community and the need for immediate action.

#### 2.4.3 Community level

##### 2.4.3.1 Involvement of local leader

Our attempt to obtain support from Sarpanch was met with resistance. He expressed doubts, citing the entrenched cultural acceptance of tobacco in the village. He suggested that our influence might be limited as outsiders and advised against pursuing the plan to avoid wasting time and effort.

##### 2.4.3.2 Involvement of shopkeepers

It was understood that the involvement of shopkeepers in this activity was pertinent, as they are one of the most critical stakeholders under COTPA. The health team visited all the shops in the village. Initially, the shopkeepers were reluctant to support the initiative, fearing that if everyone did not participate, they would lose customers and no change would happen. Awareness was raised among shopkeepers about the harmful effects of tobacco, as well as the rules governing tobacco sales and the importance of their involvement in reducing tobacco use in the area, by visiting each shop. The efforts were then channeled through the leader of the village shopkeepers’ association to bring unity in the actions taken. Even though this was not a registered association, every shopkeeper was united under the leadership of this person, who promised us support for the initiative.

Every shop in the village was revisited and the shopkeeper association leader requested the shopkeepers to abide by the rules and regulations put forward. In order to assess compliance, weekly visits were made to the shops throughout the period.

##### 2.4.3.3 At school

**Figure 1:**
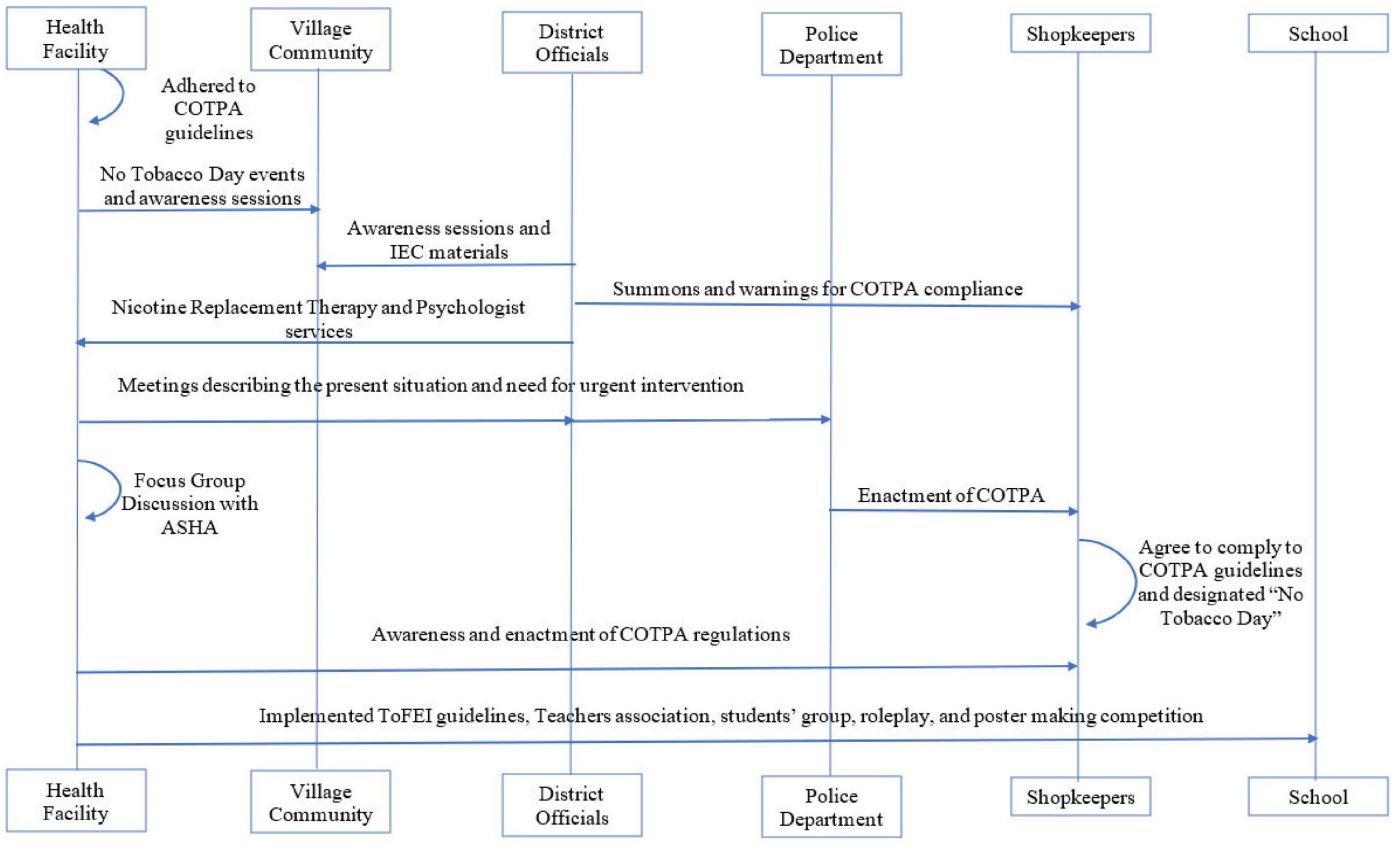
Schematic representation of the implementation plan.

The school was initially assessed with the ToFEI guidelines. The targeted activities in school were initiated with an awareness session for students, teachers, and staff. This session focused on the harmful effects of tobacco, the role of students in tobacco cessation efforts, commonly available tobacco products, how students start using tobacco, and how to stay away from tobacco. Special emphasis was given on creating a tobacco-free school campus based on the ToFEI guidelines. Students were encouraged to participate in poster-making competition and role-plays. Two weeks later, another session was organized to reinforce the ideas. The ToFEI guidelines were reassessed at the end of the activities in the school (Figure 1).

## 3 Results

### 3.1 Field findings

#### 3.1.1 Availability

Tobacco products were readily available in the village. A total of 18 shops were found selling tobacco products in this small village center. It included all types of shops like tea stalls, grocery shops and stationery stores.

#### 3.1.2 Prevalence of tobacco use

Tobacco use was very common in the village. Seven out of the 10 patients attending the Outpatient Department (OPD) of the Community Health Centre (CHC) were tobacco users. Upon conducting surveys in nearby houses, it was observed that every house had more than half of its members using one or the other form of tobacco. Tobacco usage has become a part of the culture in the area. It was a common tradition to serve them on various occasions in the villages, such as weddings and even at funeral functions.

#### 3.1.3 Type of tobacco products

A wide variety of tobacco products are being used in the area. Smokeless tobacco products, such as mava, masheri, gutka, pan, and khaini, were the most commonly used. Women predominantly consumed smokeless tobacco products, while men used bidis as well as smokeless tobacco products.

Shopkeepers gave an idea about tobacco sales in the area. Smokeless forms of tobacco account for 90% of sales. Most of the shops have an average daily sale between 200 and 300 rupees (roughly 20–30 packets). Children below the age of 18 years were coming to buy tobacco products. Students in school uniforms never came to buy tobacco products in any of the shops. The shopkeepers were unaware that tobacco products should not be sold to minors and they were also afraid of losing customers on denial to sell. The children often reported that they are buying it for their family member who were already tobacco users. But shopkeepers pointed out that while sending children to purchase tobacco was common, sometimes children exploited it to buy tobacco for themselves without getting suspicious. Such practices encouraged early initiation of tobacco use and normalized it within the families.

Some families even purchased tobacco supplies along with the regular groceries on a weekly or monthly basis. Different forms of tobacco products for the family members were purchased together depending on their age and gender—milder forms for children, smokeless ones for adults and bidis typically for men and older adults.

#### 3.1.4 Factors affecting type of tobacco use

Cost involved was the main factor affecting tobacco use pattern in the area. Since smokeless tobacco products were much cheaper, they were preferred over smoking forms by the villagers. Most men who used smoking forms of tobacco used bidis rather than cigarettes for the same reason.

It is also dependent on the age and gender. It was observed that women were using smokeless tobacco products only, while the usage in men varied with age. At a younger age, most of the men used smokeless tobacco products, while older men also used bidis and very rarely hookahs. Children were consuming milder forms of tobacco, often provided by their parents or relatives, who believed it could help in suppressing hunger.

#### 3.1.5 Adherence to tobacco-related rules and regulations

Most of these regulations were not being followed in the area. Smoking in public places was common, with one of the tea stalls serving as the central spot. Five out of the 18 shops selling tobacco products were functioning within the 100 yards circumference of the government school, thereby violating provisions under COTPA. Even the hospital premises and school premises did not adhere to the COTPA guidelines. The signage and boards were not in place, and committees were not functional in both settings.

### 3.2 Focus group discussion findings

All the nine invited ASHAs participated in the FGD.

Key verbatim from FGD illustrated the high prevalence, socio-cultural factors, and challenges in tobacco cessation efforts:

#### 3.2.1 Prevalence and patterns

*“2–4 members of every house use one or the other form of tobacco.”*

*“Even 10-year-old children use tobacco.”*

*“65% of people in my area use tobacco.”*

#### 3.2.2 Family and cultural factors

*“Parents themselves give tobacco to children.”*

*“They believe that tobacco helps in reducing hunger.”*

*“Even my husband uses tobacco and does not listen to me.*

#### 3.2.3 Barriers

*“Nobody will listen to me.”*

*“It is difficult to control at our level. Government should ban it at production level.”*

*“We fill CBAC forms, but we do not emphasize much on tobacco and alcohol use.”*

#### 3.2.4 Possible solutions

*“No use just talking to people, we can also do that. If you can provide any medicines, we will refer patients from the villages.”*

**Figure 2:**
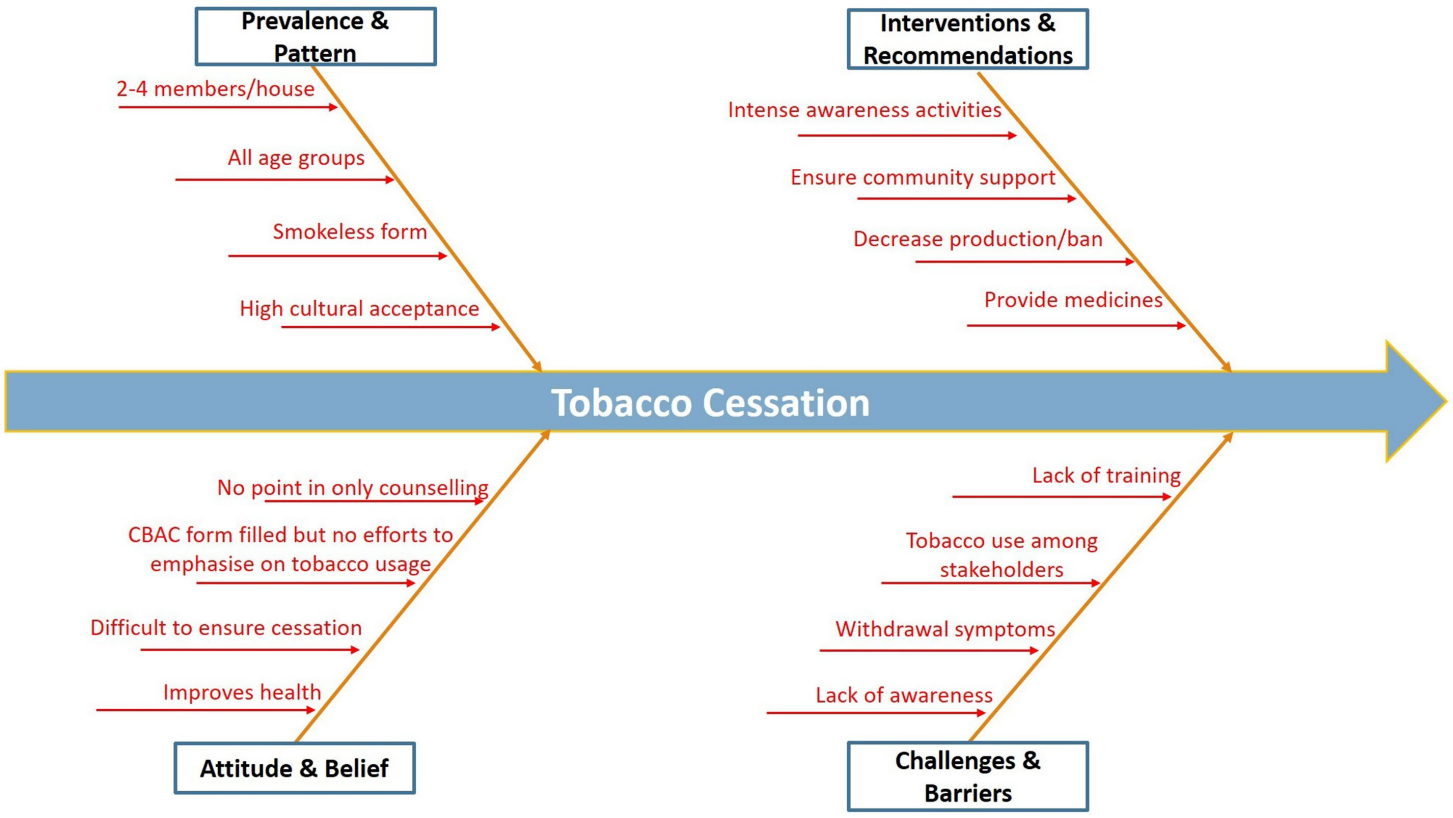
Fishbone diagram—depicting patterns, contributing factors, challenges, and recommendations related to tobacco usage in the village.

*“Nobody will listen unless a proper support or alternative is offered* (Figure 2).”

### 3.3 Outcomes of the activities

#### 3.3.1 At health facility

It was observed that the health facility did not adhere to the COTPA regulations. The use of tobacco products within the premises of health facility by patients or staff was strictly prohibited. All appropriate warning boards according to the regulations and awareness materials were placed across the hospital premises.

Every patient attending the OPD was asked about their tobacco use history and a separate register was initiated for noting this information. Brief interventional therapy for tobacco cessation was given to all those who were found using any form of tobacco. The intensity of addiction among tobacco users was assessed using the Fagerstrom Test for Nicotine Dependence (FTND) (18), and the transtheoretical model for behavior change (19) was used to determine their readiness to quit. Flip charts were used to convey the information in a patient-friendly manner.

#### 3.3.2 At the administrative level

The authorities from the No Tobacco Wing became aware of the prevailing situation in the village. They initiated regular monitoring and inspection visits to assess compliance with tobacco control regulations in the area. They assured that action will be taken against those who are found violating the legal provisions under COTPA. A proper plan was put in place, leveraging the support from the district health team to offer on-demand psychological support and ensure the availability of Nicotine Replacement Therapy at the center, aiming to address withdrawal symptoms and facilitate successful tobacco cessation journeys for individuals seeking assistance.

Police have also extended their full support for all the initiatives. They have assured active participation in enforcing the regulations and in ensuring the long-term sustainability of the initiative.

#### 3.3.3 At community level

##### 3.3.3.1 Shopkeepers

Shopkeepers agreed to implement the following measures:

- Tobacco products will not be displayed openly in the shops.
- All shopkeepers have agreed to stop the sale of tobacco products to those under 18 years of age.
- Shopkeepers within 100 yards of the school agreed to stop the sales after the current stock is over.
- A collective decision was reached to designate one day each month as the “No Tobacco Day” within the village. It was unanimously agreed that the Second Thursday of every month would be dedicated for this initiative.
- Awareness materials highlighting the harmful effects of tobacco and warnings stating that tobacco products will not be sold to individuals under 18 years were displayed at every shop in the village.

##### 3.3.3.2 At school

ToFEI related regulations were enacted in the school. Official warning boards, awareness materials, and posters made by students were pasted in the school premises. A dedicated committee of teachers was formed under the principal, and a staff was designated in charge of all the activities related to ToFEI. A student council was formed by selecting representatives from each class. They were entrusted with the responsibility of monitoring tobacco usage among their batch students and reporting it to teachers or OPD staff in the health facility. The team of selected student council members enacted the role-plays in the school assembly to generate awareness. Sustainability of the measures were ensured by coordinating the activities of the school committees with the health team (Table 1).

**Table 1:** Pre and post intervention Tobacco Free Educational Institution (ToFEI) score of the school.

S. no	Criteria	Weightage points	Points-pre intervention	Points-post intervention
1	Display of ‘Tobacco Free Area’ Signage inside the premise of Educational Institute at all prominent place(s).	Mandatory (10)	0	10
	The name/designation/contact number are mentioned/updated in the signage.	Mandatory (10)	0	10
2	Display of “Tobacco Free Education Institution” signage at entrance/boundary wall of Educational Institute.	Mandatory (10)	0	10
	The name/designation/contact number are mentioned/updated in the signage	Mandatory (10)	0	10
3	No evidence of use of tobacco products inside the premise, i.e., cigarette/beedi butts or discarded gutka/tobacco pouches, spitting spots.	Mandatory (10)	0	0
4	Poster or other awareness materials on harms of tobacco displayed in the premise.	9	0	9
5	Organization of at least one tobacco control activity during last 6 months.	9	0	9
6	Designation of Tobacco Monitors and their names, designations, and contact number are mentioned on the signages	9	0	9
7	Inclusion of “No Tobacco Use” norm in the EI’s code of conduct guidelines	9	9	9
8	Marking of 100 yards area from the outer limit of boundary wall/fence of the EI	7	0	7
9	No shops selling tobacco products within 100 yards of the Educational Institute	7	0	7
	Total	100	9	90

The score of the school increased from 9 to 90 as a result of the intervention. By attaining a score of 90%, the school became eligible for the for participation in the ToFEI Award Scheme by the Ministry of Health and Family Welfare, India.

## 4 Discussion

The present study was conducted as part of the public health grand rounds of the Department of Community Medicine and Family Medicine, AIIMS Jodhpur. By identifying the high prevalence of tobacco use among patients as well as in the community, the team developed a comprehensive strategy to reduce the use of tobacco products in the area. Most of the studies in this topic focus on identifying the prevalence and predictors leading to tobacco use. There are very few studies that deal with the efforts taken to reduce tobacco use and exposing grassroot level challenges.

The strategies used in the present study were similar to the one used in a study done in Maharashtra state of India (20). The initial step was a comprehensive situational analysis to assess the sociodemographic profile, cultural context and tobacco use patterns in the area. Widespread availability of tobacco products and high prevalence of tobacco use across all demographic groups were common in both studies. The most used tobacco products varied by age and gender. The predominant form among men was *kharra*, while women used *kharra* and *mishri.* In contrast, the present study area did not have *kharra* users. The high prevalence of smokeless tobacco use was similar in both study settings. *Mava, mishri, gutka and pan* were commonly used by the villagers in our study area. The preference for bidis among older adult men remained consistent in both the studies. Pan masala was common among adolescents. A cross-sectional study done by Gupta et al. in a rural village of Rajasthan showed higher prevalence of smokers than smokeless tobacco users among adolescents (21). Variations in the forms of tobacco used across different age groups and study settings highlight the regional differences in the type and accessibility of various forms of tobacco products across the country.

The present study was based on a multi-level approach—community based, school based, interpersonal, and health facility-based interventions. The study conducted by Chatterjee et al. also adopted intense activities at the community level, school level, and interpersonal level as its key strategy (20). Community education and mobilization were the cornerstone of these strategies, and it was achieved through trained staff in the respective villages. Comparison of the scale and resources of the present study with the study done by Chatterjee et al. highlights the feasibility of these tobacco control interventions in resource-limited settings. The present study had limited manpower and no dedicated funding. Still the planned interventions were implemented in the village through strong institutional and community-level commitment.

The Ministry of Health & Family Welfare in India has given out the standard operating procedures for making villages tobacco-free (22). This guideline, published in 2024, emphasizes strategies like implementing ToFEI in all schools, forbidding the sale of tobacco products to those under the age of 18 years, displaying warning signs in prominent areas, and ensuring strong institutional and political commitment. These strategies used in the present study align closely with this national guideline. The study done by Gupta et al. has also highlighted the need to emphasize school-based awareness programs by including teachers, parents and peer groups to tackle the rise in prevalence of tobacco use among adolescents. This study underscores the need to adapt a tailor-made combination of these strategies that are sensitive to the socio-cultural and demographic factors of the area. It also provides critical insights into the challenges encountered in implementing them at the grassroots levels.

## 5 Challenges faced

### 5.1 Resistance from local leader

Community leaders, including local politicians or influential figures, can resist tobacco control efforts for various reasons. These local leaders may have personal or economic benefits from production or sales of tobacco products. They may perceive tobacco control as a low-priority issue compared to the other pressing issues in the area. It is important to overcome this resistance by demonstrating the public health benefits of tobacco cessation initiatives and building a good relationship between health systems and the leaders.

### 5.2 Cultural acceptance

Tobacco use was deeply ingrained in the community’s cultural practices. Tobacco had a very significant social, ceremonial, or symbolic value in the area. It is being used in social gatherings and rituals, serving as a symbol of hospitality, respect, and unity within the community. This widespread acceptance made it very challenging to promote cessation efforts in the area.

### 5.3 Resistance from shopkeepers

Economic concerns refrained shopkeepers from supporting the initiative. Tobacco products often contribute a significant portion of their revenue, and reducing or eliminating sales could impact their livelihood. Additionally, some feared losing customers if they complied with regulations or community-led initiatives aimed at restricting tobacco sales. This resistance was overcome by engaging with shopkeepers, highlighting the long-term benefits, and ensuring that every shopkeeper compiled with the guidelines to prevent shops from losing customers to others.

### 5.4 Behavioral constraints

The credibility of anti-tobacco initiatives is questioned when the key stakeholders like government officials, hospital staff, and school teachers, are themselves tobacco users. These behaviors can also result in decreasing the public trust in these initiatives and act as a barrier to effective implementation.

Students raised concerns about certain teachers using tobacco products during class time. Our team engaged in discussions with the teachers regarding this issue and provided counseling. The teachers pledged to decrease their tobacco consumption and refrain from using it on school premises.

## 6 Strengths

The study draws attention to critical issues like widespread tobacco use, lack of awareness, tobacco use among minors and women, and weak implementation of regulations. The main strength lies in the strong community-based approach with active involvement of multiple stakeholders. Integrating tobacco cessation counseling into routine OPD services showed how preventive efforts can be incorporated into existing health systems with the available resources. Lessons learned from this study can be replicated in similar settings after local adaptations.

## 7 Limitations

This was a service-based initiative and many activities were based on practical convenience rather than formal research methods. Precise estimates were not made as much of the information was collected through informal discussions. The reliance on self-reporting introduces the possibility of underreporting and reporting bias. Follow-up studies are important to identify the long-term impact of the strategies used.

## 8 Way forward

Long-term sustainability: The most important aspect of any tobacco cessation effort is to ensure sustainability. Only sustained effort can lead to long-lasting behavioral changes within the community. Opportunities for collaboration, funding, and community ownership for maintaining the program activities beyond the initial phase should be explored.

Enacting COTPA in other schools of the village.

Further assessment of COTPA and ToFEI guidelines across the Jodhpur district and a plan of action for strengthening the enforcement of the same, in coordination with the ‘No Tobacco Wing’.

Integration with healthcare services: Integrating tobacco cessation services into existing healthcare services can improve the accessibility and acceptability of these services. This can also ensure the easier and faster delivery of services using existing infrastructure.

Tailored interventions: Interventions should be tailored to the target population. Aspects such as language, traditional beliefs, and socioeconomic factors should be taken into consideration while developing an intervention. This can ensure the relevance and effectiveness of any interventions given.

Capacity building: Empowering local stakeholders can enable them to support individuals in quitting tobacco effectively. Training should be provided through targeted capacity-building programs for all concerned stakeholders including local healthcare workers and community volunteers.

**Figure 3:**
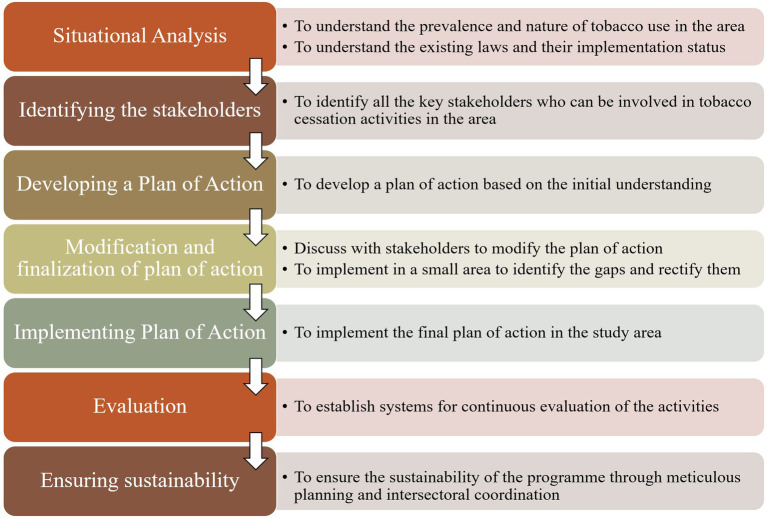
Framework for tobacco cessation activities in similar settings.

Policy advocacy: There is a need to advocate stronger enforcement and better compliance with tobacco control policies at every level. The interdisciplinary collaboration of various stakeholders is vital for better implementation of the existing laws. Sustained policy advocacy informed by community insights can help bridge the gap between policy and practice. Special focus should be placed on rural and underserved communities where the implementation gaps are often greatest (Figure 3).

## 9 Conclusion

Despite the existing rules and regulations, tobacco remains a major cause of morbidity and mortality. Rural communities bear a disproportionate burden of the harmful effects of tobacco. This is due to the lack of awareness and limited enforcement of the existing regulations. Addressing this issue requires a multifaceted approach focused on creating awareness and strengthening community engagement. Key components of these efforts should also include capacity building, active stakeholder involvement, and strong intersectoral coordination. This can lead to effective reinforcement of these existing mechanisms related to tobacco control. The interventions should also be tailor-made, considering the cultural differences of the area, and sustainability must be ensured to achieve an impactful tobacco control. It is high time to take collective action to address this important public health challenge and safeguard the wellbeing of communities.

## Data Availability

The original contributions presented in the study are included in the article/supplementary material, further inquiries can be directed to the corresponding author.
